# Decoupling the refractive index from the electrical properties of transparent conducting oxides via periodic superlattices

**DOI:** 10.1038/srep33006

**Published:** 2016-09-13

**Authors:** David Caffrey, Emma Norton, Cormac Ó Coileáin, Christopher M. Smith, Brendan Bulfin, Leo Farrell, Igor V. Shvets, Karsten Fleischer

**Affiliations:** 1 School of Physics, Trinity College Dublin, Dublin 2, Ireland; 2 Advanced Materials Bio-Engineering Research Centre (AMBER), Trinity College Dublin, Dublin 2, Ireland; 3 Centre for Research on Adaptive Nanostructures and Nanodevices (CRANN), Trinity College Dublin, Dublin 2, Ireland; 4 KSU-Aramco Center, King Saud University, Riyadh 11451, Saudi Arabia; 5 School of Physics, Beijing Institute of Technology, Beijing 100081, P. R. China; 6 Institute of Solar Research, German Aerospace Center (DLR), 51147 Köln, Germany

## Abstract

We demonstrate an alternative approach to tuning the refractive index of materials. Current methodologies for tuning the refractive index of a material often result in undesirable changes to the structural or optoelectronic properties. By artificially layering a transparent conducting oxide with a lower refractive index material the overall film retains a desirable conductivity and mobility while acting optically as an effective medium with a modified refractive index. Calculations indicate that, with our refractive index change of 0.2, a significant reduction of reflective losses could be obtained by the utilisation of these structures in optoelectronic devices. Beyond this, periodic superlattice structures present a solution to decouple physical properties where the underlying electronic interaction is governed by different length scales.

A medium’s refractive index (n) describes how the oscillating electric field of a light wave causes the disturbance of electrons which subsequently emit light. This emitted light will be out of phase with the original incident wave and cause interference. This results in an apparent reduction of the rate of propagation of light through the medium and hence a refractive index >1. The Fresnel equation describe that light incident upon an interface experiences reflection proportional to the refractive index mismatch of the two mediums. Thus matching of the refractive index of successive media is important if transmission is to be maximised.

Changing the refractive index of a medium as a whole is possible via temperature, mechanically induced strain, piezoelectricity and the introduction of porosity into the material^1^. Despite this, for typical optical applications, it is easier to replace a material with one of higher or lower refractive index rather than modifying the refractive index of a given material. The situation is more complex if the choice of material is governed by more than just the refractive index.

This is most apparent in the current generation of Transparent Conducting Oxides (TCOs), where the key requirements are high optical transparency and electrical conductivity. Only a small number of employable materials are currently known. Thus, the selection of a TCO with an optimal refractive index for a given device is often not feasible. This leads to significant unwanted reflection losses at the TCO and its interfaces in transparent devices, such as solar cells or displays. Developing a strategy to tune a TCO’s refractive index is important to improve many optoelectronic devices.

Modifying the refractive index while retaining the conductivity and mobility is difficult as both properties are intertwined with the electron density of the material. One of the leading methodologies for the tuning of *n* is cation substitution, wherein atoms of a material are replaced by those of a lower electron density material. This results in an overall reduction of the electron density of the medium and hence, the refractive index.

An example of such is ZnO:Al, one of the leading low cost alternatives to Indium Tin Oxide (ITO)^2^,^3^,^4^,^5^,^6^. It has been demonstrated that by substituting controlled amounts of Mg for Zn in ZnO:Al to form Zn_1−*x*_Mg_*x*_O:Al the refractive index and optical bandgap of the material can be tuned^7^,^8^,^9^,^10^,^11^,^12^,^13^,^14^,^15^. Manipulation of the refractive index via cation substitution comes with significant repercussions. Increases in neutral impurity scattering originating from the implanted dopants leads to a reduction in mobility and conductivity of the films^7^,^9^,^11^ and the high cation inclusion levels required can lead to dephasing^7^,^9^. These detrimental effects mean that an alternative method of tuning the refractive index without loss of the electrical properties is highly desirable.

In this work, we demonstrate the use of a superlattice composed of alternating thin pseudo 2D layers of a TCO and a lower electron density material. The confinement of the low refractive index material to layers substantially thinner than the wavelength of visible light results in a structure which behaves electronically as a set of successive conductive layers and optically as a medium equivalent to a mixture of the constituent materials. Thus the refractive index is effectively decoupled from the electrical properties, allowing for substantial changes while maintaining the high qualities of the TCO. This concept is illustrated in Fig. 1.

Oxide superlattices themselves have been previously well studied for other properties. Naturally occurring structures in layered oxides can induce variation in properties such as the photoluminescence^16^, crystalline quality^17^ and thermoelectric properties^18^,^19^. Artificial superlattices can optimise, enhance or tune the optical bandgap^20^, oxidisation states^21^, resistive switching^22^, thermoelectric properties^23^,^24^,^25^,^26^, TFTs^27^ and ferroelectric properties^28^,^29^,^30^,^31^.

Prominent n-type TCOs, amorphous InGaZnO_4_ (a-IGZO) and ZnO:Al, were selected as the TCO layers alongside SiO_*x*_ as the low refractive index layer. ZnO:Al was selected as a TCO layer in order to facilitate direct comparison with previously mentioned results obtained via cation substitution of Mg. a-IGZO was selected as a result of its nature as a high mobility n-type TCO, with a number of properties contrasting with those of ZnO:Al. These include the nature of the crystallographic order of the materials (amorphous vs. crystalline) and the variation in their carrier generation mechanisms (intrinsic vs. extrinsic)^3^,^32^,^33^.

SiO_*x*_ was selected for its cost effectiveness and low refractive index (*n* ≈ 1.45) to facilitate tuning over a wide range of values. Increasing *n* by inclusion of dielectric layers with higher refractive index than the host TCO is equally feasible to the reduction of *n* described in this work.

Beyond this, modifications such as combining high carrier concentration doping layers with high mobility transport layers are theoretically possible by employing this concept. In addition, the above could be combined with the inducement of optical band gap shifts (as shown for a-IGZO by Abe *et al*.^20^) by selection of appropriate periodicity and functionalisation layer for confinement effects.

## Results

### Properties of a TCO/Dielectric superlattice

**Figure 2:**
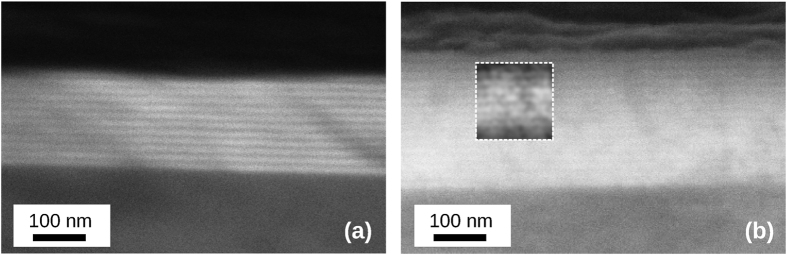
Cross-sectional SEM of of TCO/SiO_*x*_ superlattices. Cross sectional image of (**a**) 10/7.5 nm a-IGZO/SiO_*x*_ and (**b**) 10/5 nm ZnO:Al/SiO_*x*_ superlattices on glass substrate (**a**) was obtained using a backscatter electron detector and (**b**) with a secondary electron detector. Inset of (**b**) has been post processed to locally enhance contrast within the layered structure.

The deposited superlattices consisted of 10 nm TCO layers (either a-IGZO or ZnO:Al) interspersed with SiO_*x*_ layers of thickness 2–10 nm. The periodic nature of the layered structure of the superlattices was confirmed via cross-sectional Scanning Electron Microscopy (SEM). Representative images of the superlattices deposited in this work are provided in Fig. 2. X-Ray Reflectivity (XRR) measurements were fitted to obtain the average individual layer thickness of the deposited films. Further details of the characterisation of the deposited superlattices are given in the supplementary information including fits of the XRR data (Supplement S1 and S7). X-Ray Diffraction (XRD) of the superlattices demonstrated the a-IGZO to be amorphous and ZnO:Al to be crystalline for all samples (see Supplement S2).

**Figure 3:**
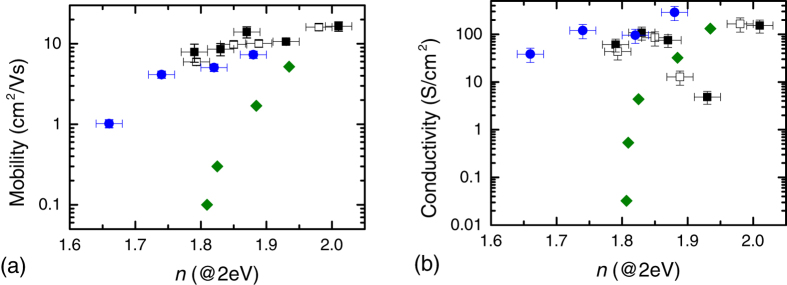
Mobility vs. *n* of the deposited Superlattices. Modulation in the (**a**) mobility and (**b**) conductivity of the superlattices plotted against their refractive index at *hν* = 2 eV (*λ* = 620 nm). Results for cation substituted Zn_1−*x*_Mg_*x*_O:Al are included for comparison. (■) a-IGZO/SiO_*x*_ superlattices on glass substrate, (□) a-IGZO/SiO_*x*_ superlattices on Al_2_O_3_, (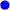) ZnO:Al/SiO_*x*_ superlattices, (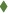) Zn_1−*x*_Mg_*x*_O:Al grown by spray pyrolysis from Fleischer *et al*.^7^.

The mobility and conductivity of the a-IGZO and ZnO:Al superlattices are plotted as a function of refractive index in Fig. 3. Values obtained previously for cation substitution in Zn_1−*x*_Mg_*x*_O:Al^7^ are plotted for comparison. The refractive index was taken in the middle of the visible range (2 eV) for all samples. The refractive index of the films was found to decrease systematically with increasing SiO_*x*_ layer thickness, for both a-IGZO/SiO_*x*_ (1.97 to 1.75) and ZnO:Al/SiO_*x*_ (1.88 to 1.66) superlattices, while maintaining high mobility and conductivity. The robustness of the method was demonstrated by the consistency in the mobility and conductivity between superlattices deposited on standard microscope slides and those deposited on polished sapphire. This illustrates that no special choice of smooth substrate is required for the efficacy of the superlattices.

It should be noted that we found that, for our deposition conditions, the atomic composition of a thin a-IGZO layer (10 nm) slightly differs from a thicker one (100 nm). However, this small change did not alter the mobility of these films. Within the superlattice the composition of the individual a-IGZO layers does not vary and is similar to a single 10 nm film. Full details of the compositional analysis are given in supplementary information (Supplement S3). As can be seen from Fig. 3(a), both a-IGZO/SiO_*x*_ and ZnO:Al/SiO_*x*_ superlattice structures are found to maintain significantly higher mobility with tuned refractive indices than for equivalent films obtained previously with cation substitution of ZnO:Al with Mg^7^. In Fig. 3(b), a similar improvement is seen in the conservation of the conductivity of the superstructures, with a reduction of less than an order of magnitude observed in both cases, while cation substitution exhibited significantly greater reductions with a narrower tunable refractive index range.

On a macroscopic scale it is intuitive that structures consisting of layered conductive and non-conductive materials will lead to a significant increase in the out of plane serial resistance of the structure, resulting in a conduction anisotropy *σ*_*x*,*y*_ > *σ*_*z*_. However, on the nano-scale such assumptions can be deceptive and the situation becomes more complicated as the thickness of the individual layers within the superstructure are reduced towards the order of the electron mean free path. In such cases, this will not solely depend on the resistivity of the material but also on the nature of its bandstructure, in particular the conduction band offset relative to the TCO layers, allowing for further optimisation from an electrical point of view. As a result, in low dimensional superlattices consisting of conductive and non-conductive layers it is important to establish that conduction occurs throughout the superstructure. Should the current be unable to penetrate into the lower layers of the superlattices a sharp decrease in the conductivity with increasing SiO_*x*_ layer thickness would be expected as the conduction is limited to the topmost TCO layers. This is not seen in our samples. By comparing two 10/10 a-IGZO/SiO_*x*_ superlattices of different overall thickness (90 nm and 190 nm) and a reference 10 nm a-IGZO film this was further confirmed. A full discussion of these samples and their characterisation can be found in the supplementary information (Supplement S3).

Finite Element Method (FEM) simulations were performed to assess under which condition the current density would be homogeneously distributed over all individual TCO layers. It was found that for the thickest SiO_*x*_ layers (10 nm) this is achieved for SiO_*x*_ resistivities of less than 10^6^ Ωcm. For highly resistive SiO_*x*_ the current would be partially limited to the first few TCO layers. This simulation underestimates the current injection in two notable ways; firstly, it does not take into account non-classical effects, which are expected once the SiO_*x*_ layer thickness approaches the electron mean free path. In this case tunneling should increase the current injection. Secondly, there is evidence that the deposited SiO_*x*_ is understoichiometric and defective, making it less insulating than bulk SiO_2_^34^. A full discussion of the FEM simulations can be found in the supplementary information (Supplement S5). From these simulations and the above experimental observations we can conclude that the current is distributed throughout the superstructure. The discussions above highlight that careful choice of material is key for attainment of optimal electrical properties of the superlattices.

**Figure 4:**
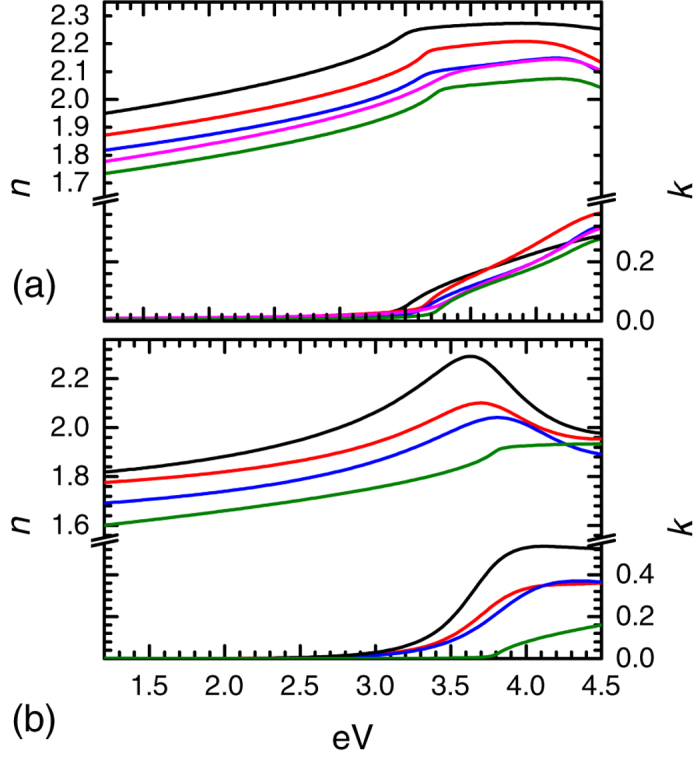
Refractive index of the superlattices. Dielectric functions of the (**a**) a-IGZO/SiO_*x*_ and (**b**) ZnO:Al/SiO_*x*_ superlattices. (–) bulk film, (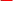) 10/**2**.**5** **nm**, (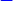) 10/**5** **nm**, (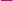) 10/**7**.**5** **nm**, (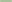) 10/**10** **nm**.

**Figure 5:**
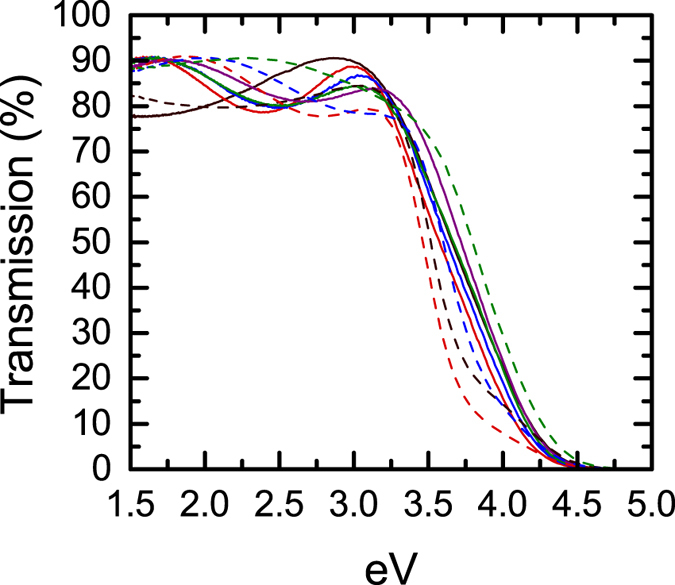
Transmission of the a-IGZO and ZnO:Al based superlattices. (—) 100 nm a-IGZO, (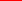) 10/**2**.**5** **nm** a-IGZO/SiO_*x*_ superlattice, (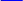) 10/**5** **nm** a-IGZO/SiO_*x*_ superlattice, (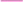) 10/**7**.**5** **nm** a-IGZO/SiO_*x*_ superlattice, (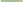) 10/**10** **nm**. a-IGZO/SiO_*x*_ superlattice, (---) 80 nm ZnO:Al, (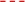) 10/**2**.**5** **nm** ZnO:Al/SiO_*x*_ superlattice, (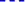) 10/**5** **nm** ZnO:Al/SiO_*x*_ superlattice, (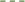) 10/**10** **nm** ZnO:Al/SiO_*x*_ superlattice.

The effective refractive indices of the superlattices are given in Fig. 4. In both cases a clear reduction of the real part of the refractive index of the superlattice is observed with increasing SiO_*x*_ layer thickness. The range over which the refractive index could be tuned was expanded relative to cation substitution. Via the use of superlattices a Δ*n* ≈ 0.2 is observed in our films with a reduction of less than an order of magnitude in the conductivity, whereas in cation substitution the change was limited to Δ*n* ≈ 0.13 accompanied by a several orders of magnitude degradation of the conductivity. The transmission spectrum of the superlattices is plotted in Fig. 5. Transmission in the visible spectrum remains in the 80–90% range for all samples, with variation originating from changes in the Fabry-Pérot oscillations due to slight thickness deviations and, more significantly, the refractive index change.

Concerning possible electronic confinement effects within the IGZO layers, we observed in both sets of superlattices some shifts in the position of the fitted bulk critical point which can be related to the band gap energy. It has been previously shown that the a-IGZO/SiO_2_ band offset can lead to strong confinement^35^, although it would be expected that thinner a-IGZO layers than used in this work would be required to induce quantisation^20^. Due to alterations in the shape of the high energy extinction coefficient (*k*) complicating analysis, it is difficult to explicitly interpret these critical point shifts as an indication of the alteration of the optical band gap. This is further discussed in the supplementary information (Supplement S6).

In this work SiO_*x*_ was employed as the low index layer due to its comparatively low index ≈1.45 and industrial relevance. However, the concept of engineering the refractive index by using superlattice structures is largely material independent. Many TCO-functional layer combinations are yet to be explored. For instance the range of tunability of the refractive index can be improved upon by replacing SiO_*x*_ with a material of lower electron density. An example of such would be MgF_2_ (*n* ≈ 1.37) a transparent material currently employed as an anti-reflective layer on crown glass^36^,^37^. The use of such a low index layer would facilitate tuning of the refractive index over a wider range than that described above.

### Estimated impact of superlattice structures in devices

With the demonstrated tunability we can assess improvements in the light harvesting of a thin film solar cell by the same method as outlined previously by Fleischer *et al*.^38^. By utilising the dielectric function calculated in this work for the a-IGZO/SiO_*x*_ superlattices and assuming a similar structure could be grown for a high refractive index material, we can calculate the estimated reflective improvement using the Fresnel equations in a transfer matrix calculation. For the glass/FTO/a-Si/Al thin film solar cell considered there, the use of the tuned TCO superlattice can modestly increase the conversion efficiency by ~2%. If our tuning concept is further developed to high index materials e.g. by using TiO_2_/IGZO superlattices and assuming that a similar structure can be grown, the refractive index could be expected to increase up to ~2.3. This would reduce internal reflections in the discussed solar cell structure at the TCO/absorber interface increasing conversion efficiency by up to 9%. By combining both internal high index and low index anti-reflective TCO superlattices improvements of up to 11% could be obtained.

In much simpler structures such as conductive transparent coatings (e.g. in displays) the overall reflection from the TCO layer can also be reduced employing the superlattice concept. For example the reflectance of a typical 500 nm ZnO:Al film on glass can be reduced from average values in the visible range of 10% to 7% and 6% using a 10/5 or 10/10 ZnO:Al/SiO_*x*_ superlattice respectively.

### Anomalous behavior of low SiO_*x*_ layer thickness a-IGZO/SiO_*x*_ superlattices

**Figure 6:**
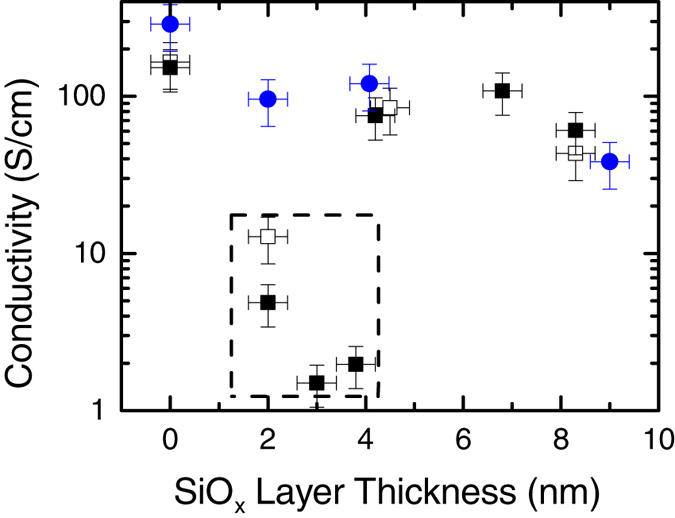
Conductivity of the superlattices as a function of the SiO_*x*_ layer thickness. Conductivity vs. SiO_*x*_ layer thickness of the a-IGZO/SiO_*x*_ superlattices and ZnO:Al/SiO_*x*_ superlattices. (■) a-IGZO/SiO_*x*_ superlattices on glass substrate, (□) a-IGZO/SiO_*x*_ superlattices on Al_2_O_3_, (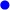) ZnO:Al/SiO_*x*_ superlattices. The low thickness SiO_*x*_ layers exhibiting lower conductivities are highlighted by the dashed box.

It can be observed in Figs 3 and 6 that some superlattices experience an anomalous decrease in conductivity. This occurs in a-IGZO superlattices with an SiO_*x*_ layer thickness below 4 nm. XRR and XRD of the thin films confirm that increased roughness at low thicknesses is not a factor. This is intuitive as such would be expected to enhance conductivity due to contact between successive a-IGZO layers. The origin is thus likely to be some electronic property of the a-IGZO or SiO_*x*_ at developed at low thicknesses. Direct measurement of the origin of this behavior would require *in*-*situ* analysis of the properties of the SiO_*x*_ layers during growth, as exposure to atmospheric conductions introduces contaminants that either compensate for or mask the effect. Such methods were not available during the growth of these superlattices and as such, we must rely on indirect evidence to gain insight into the nature of this effect.

**Figure 7:**
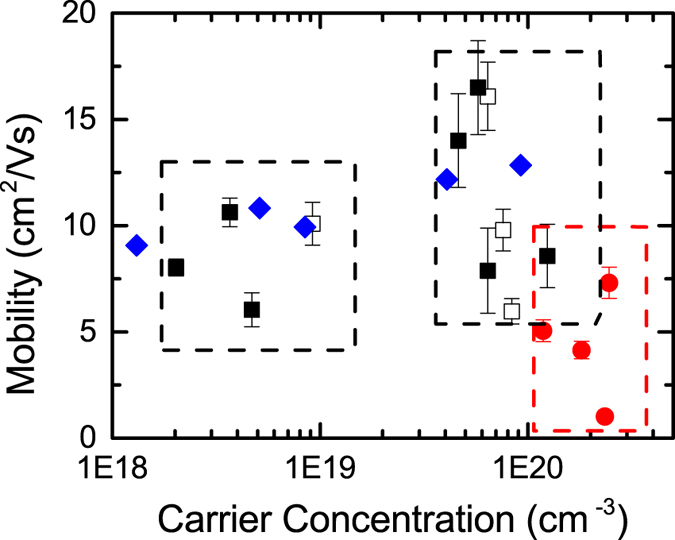
Mobility vs. carrier concentration. Variation in the carrier concentration of the superlattices with SiO_*x*_ layer thickness. (■) a-IGZO/SiO_*x*_ superlattices on glass substrate, (□) a-IGZO/SiO_*x*_ superlattices on Al_2_O_3_, (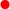) ZnO:Al/SiO_*x*_ superlattices, (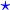) Values for bulk a-IGZO from Akihiro Takagi *et al*.^39^. The behaviour of the films can be categorised into three regions. Region A consists of the a-IGZO superlattices with SiO_*x*_ layers of 2–4 nm thickness. Region B consists of the a-IGZO superlattices with SiO_*x*_ layers of 4–10 nm thickness. Region C consists of the a-ZnO:Al superlattices. Variation of the mobility within these regions is a result of the modulation of the SiO_*x*_ thickness over the specified range.

Significant variation is observed in the carrier concentration of the films. Figure 7 depicts the mobility of the superlattices as a function of their carrier concentration alongside results obtained for standard a-IGZO films deposited at varied oxygen partial pressures by Takagi *et al*.^39^. Two distinct regions can be identified. Region A, consisting of superlattices of SiO_*x*_ layer thickness >4 nm, and region B, consisting of those of <4 nm alongside the bulk a-IGZO films. It can be noted that the ZnO:Al/SiO_*x*_ superlattices, as shown in Figs 6 and 7 (region C), do not show these variations. In order to understand the possible origins of this we can address the fundamental carrier generation mechanisms of ZnO:Al and a-IGZO. The majority carriers in ZnO:Al are known to originate from the extrinsic substitution of Al on the Zn site^40^,^41^. In contrast, the donor defect in a-IGZO is widely considered to be strongly tied to intrinsic oxygen vacancy defects, resulting in the carrier concentration being oxygen sensitive^39^,^42^,^43^,^44^. As a result, while both materials are sensitive to oxygen within their structure, due to oxygen interstitials acting as electron pinning defects, it is expected that the a-IGZO would show a significantly higher sensitivity. Thus by considering the similarity of the behavior of the a-IGZO to that deposited at varying partial pressures of oxygen and the difference in the behavior of the ZnO:Al and a-IGZO superlattices, we tentatively ascribe this behavior to variation in the oxygen environment during growth or in the deposited superlattices.

We believe that such variation in oxygen could be explained by the properties of low thickness SiO_*x*_. It has been demonstrated that the formation energy of defect can be enhanced for thin SiO_*x*_ layers as deposited here^34^,^45^,^46^. We theorise that the root cause of the observed changes in the a-IGZO layers is most likely an altered defect density for ultrathin SiO_*x*_ layers leading to a locally changed oxygen structure. This could take the form of variation in the local oxygen partial pressure near the surface of the growing film or enhanced oxygen/cation interlayer diffusion induced by the under-stoichiometric SiO_*x*_ surface. This diffusion could take the form of either Si diffusion, which has been shown to act as a carrier suppressor in amorphous InZnO and IGZO^47^,^48^, or oxygen vacancies, which are heavily tied to the donor defect structure of a-IGZO^39^,^42^,^43^,^44^.

We thus believe that the indirect evidence as outlined above supports the conclusion that the change in the electrical properties of the films is related to the formation of defective SiO_*x*_ at low thicknesses. In order to verify this a precise methodology for *in*-*situ* measurement of the properties of the interface during growth, such as combination of *in*-*situ* XPS and magnetron sputtering, would be required.

## Conclusion

a-IGZO/SiO_*x*_ and ZnO:Al/SiO_*x*_ superlattices were deposited to decouple the value of the refractive index of the films while maintaining the optoelectronic properties.

By varying the thickness of the periodic SiO_*x*_ layers the refractive index of a-IGZO and ZnO:Al were tuned by Δ*n* ≈ 0.2. Over this refractive index change, the mobility for the a-IGZO and ZnO:Al superlattices varied from 16.5 cm^2^/Vs to 6 cm^2^/Vs and 7.3 cm^2^/Vs to 1 cm^2^/Vs respectively. Conductivity was found to be maintained within an order of magnitude of the original values. Selection of an appropriate tuning material is key to attaining strong conservation of the electrical properties and it is believed that by careful choice of refractive index tuning layer this methodology could be expanded to other materials to produce further optimised results in terms of conductivity and refractive index.

In terms of possible applications, we calculated that a reduction of 10% to 6% in reflected losses are expected if this technique is applied to transparent conducting optical coatings.

We believe that the discussed results demonstrate the great potential for this superlattice based technique, not just to dissociate the optical and electrical properties, but also for the decoupling of other physical quantities where the atomic interactions are governed by different length scales.

## Methods

### Sample preparation

Samples were deposited via Radio Frequency (RF) magnetron sputtering via co-sputtering from separate 2 inch diameter InGaZnO (a-IGZO), ZnO:Al (2 at% Al) and SiO_2_ targets. Targets were sputtered sequentially in order to produce a layered structure. 100 W of power was applied to the a-IGZO and SiO_2_ targets during deposition while ZnO:Al was sputtered using 50 W. Power to the TCO target was ramped down between successive layers in order to maintain a consistent composition throughout the superlattice. Nominal elemental composition of the a-IGZO target was 1:1:1:4 (In:Ga:Zn:O) as confirmed by Energy Dispersive X-ray (EDX) spectroscopy. The sputtering chamber was evacuated to below 10^−2^ Pa prior to deposition, then backfilled with 0.45 Pa of Ar during sputtering. Films were deposited onto Menzel-Glaser glass microscope slides and polished c-plane aligned sapphire supplied by CrysTec. Substrates were ultrasonically cleaned in acetone and isopropanol prior to deposition. Samples were deposited at a substrate temperature of 450 °C. This temperature was selected to maximise conductivity, while maintaining the amorphous nature of the a-IGZO. Growth rates of the films were determined via XRR and ellipsometry measurements of representative samples. Superstructures of varying SiO_*x*_ layer thickness were deposited by controlling the SiO_*x*_ layer growth time. Superlattices were grown to a total thickness of 150–200 nm in order to facilitate optical comparison between samples. Films were capped with 10 nm of the TCO layer in order to facilitate electrical contacting. The structure obtained for each superlattice can be found in the supplementary information (Supplement S7).

### Post Treatment

Films were annealed in a nitrogen atmosphere at 320 °C for highest conductivity, with properties being measured *in situ* via 4 point probe in linear configuration. The typical improvement in conductivity of the films was approximately an order of magnitude in both cases.

### X-Ray-Diffraction

XRD patterns have been measured with a *Bruker D8 Advance* using a Mo-K*α* source. Crystal phase and orientation of the ZnO:Al/SiO_*x*_ superlattices were obtained using *Bruker*’s EVA program. Crystallite size was obtained using the Scherrer equation.

### X-Ray-Reflectivity

X-Ray Reflection (XRR) measurements obtained with a *Bruker D8 Discover* equipped with a monochromised Cu-K*α* source were fitted using the *Bruker* Leptos program in order to obtain the average thickness of the TCO and dielectric layers and density of the films.

### Scanning Electron Microscopy

Cross-sectional Scanning Electron Microscopy (SEM) was performed using a *Carl Zeiss Ultra*. Samples were cleaved prior to scanning in order to present a clean edge for imaging. All images were obtained at 5 keV to enhance the signal from the surface. The a-IGZO/SiO_*x*_ superlattices were imaged using the backscatter electron detector which produced a high contrast due to the significant variation in Z between a-IGZO and SiO_*x*_. The ZnO:Al/SiO_*x*_ superlattices were imaged using the secondary electron detector. Images of the ZnO:Al/SiO_*x*_ superlattices were complicated by the comparitively low Z contrast between layers. To compensate for this, post-processing was performed on a portion of the image to smooth and maximise contrast, as shown in the inset of Fig. 2(b). Unfortunately there is additional contrast within the ZnO:Al layers due to their polycrystalline nature. This results in a rougher cleavage plane than that obtained for the a-IGZO film.

### Optical Analysis

UV Visible spectrophotometry (*Lambda 650S Perkin Elmer*) was used to measure the transmission and reflectance of the films. Films were scanned from 1.5–5 eV. The absorption coefficient (*α*) was calculated from the transmission and reflectance data using equation 1.


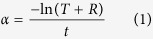


where *T* and *R* are the transmission and reflectance of the films respectively at a given energy and *t* is the thickness of the films. Tauc plots of *α*^−*n*^*hν* vs. *hν*, where n = 1/2 for direct band semiconductors, were performed on a-IGZO with sapphire substrates in order to determine the optical band gap. Tauc plots of the samples deposited on glass were complicated by the coincidence of the onset of absorption of the TCO and glass substrate. The refractive index was obtained via variable angle spectroscopic ellipsometry (*Sopra GESP 5*). Measurements were obtained at angles close to the pseudo Brewster Angle of 62, 65 and 68 for a-IGZO and 59, 62, 65 for ZnO:Al. The superstructures were modeled as an effective medium with a critical point and UV oscillator to accurately describe the high transmission and onset of absorption. The refractive indices of the a-IGZO/SiO_*x*_ superlattices were modeled by use of a 3D critical point alongside a UV oscillator fixed at 5 eV, while for the ZnO:Al/SiO_*x*_ superlattices a 1D critical point alongside a UV oscillator was employed. Values of the overall thickness of the superlattices obtained from the ellipsometry fits were found to be consistent with those obtained via XRR further confirming the validity of the model. Decoherence in the reflected light resulting from increased roughness at the ZnO:Al/SiO_*x*_ interface leads to a significant reduction in the resolution of our measurement in the high energy range for the 10/10 ZnO:Al/SiO_*x*_ superlattice. Refractive index of the bulk a-IGZO and ZnO:Al were found to be increased and reduced respectively relative to database values due to density variations present in the samples (See discussion of XRR in Supplement S1 for details).

### Electrical characterisation

Mobility and conductivity of the samples were obtained via Hall measurements performed using silver adhesive contacts arranged in a Van der Pauw configuration in conjunction with a 10 T field in a Physical Property Measurement System (*Quantum Design*). Forward and reverse bias measurements were taken in all directions to minimise any geometry or sample inhomogeneity based errors. Electrical properties, such as conductivity, were calculated using the overall thickness of the superlattice, including the SiO_*x*_ layers. Carrier concentration was calculated using only the combined thickness of the TCO layers.

## Additional Information

**How to cite this article**: Caffrey, D. *et al*. Decoupling the refractive index from the electrical properties of transparent conducting oxides via periodic superlattices. *Sci. Rep.* **6**, 33006; doi: 10.1038/srep33006 (2016).

## Supplementary Material

Supplementary Information

## Figures and Tables

**Figure 1:**
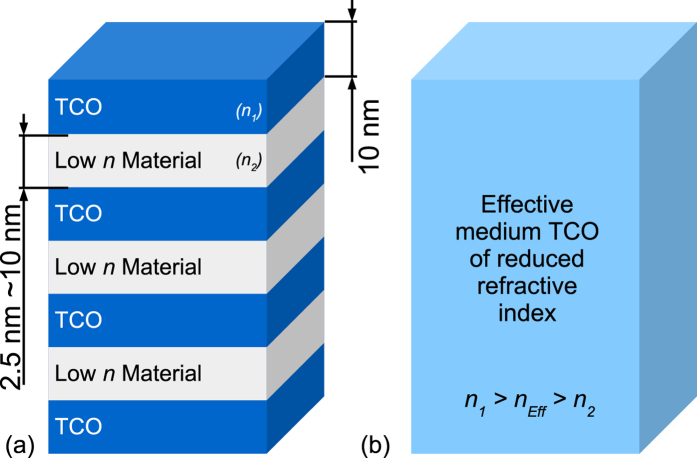
Conceptual Schematic of Superlattice. (**a**) The structure of the superlattices. (TCO layers (n_1_) of 10 nm interspersed with low *n* (n_2_) layers of 2.5 nm to 10 nm thickness). (**b**) The optical response of the superlattice, namely an effective medium of refractive index index n_1_ > n > n_2_.
