# Supplementary material for: Decoupling the refractive index from the electrical properties of transparent conducting oxides via periodic superlattices
Source: Sci Rep. 2016 Sep 13;6:33006. doi: 10.1038/srep33006 (PMC5020692; doi:10.1038/srep33006)
Supplement: Supplementary Information [file srep33006-s1.pdf]

Decoupling the refractive index from the electrical properties of transparent conducting oxides via periodic superlattices

David Caffrey, Emma Norton, Cormac Ó Coileáin, Christopher M. Smith, Brendan Bulfin, Leo Farrell, Igor V. Shvets, and Karsten Fleischer

Supplementary Information

S1. X-Ray Reflection measurements of the superlattices

X-Ray Reflectivity (XRR) analysis has previously been demonstrated to be an invaluable tool in the investigation of superlattice properties[1–5]. Here XRR was employed to confirm the thickness of the periodic layers, interface roughness and to obtain the density of the deposited material. The densities of the materials were obtained from measurements of representative samples of the films. The density of the deposited a-IGZO was found to be 6.8g/cm^3, higher than typically reported values[6]. This densification of the material can be attributed to the high level of indium enrichment of the films (see Supplement S3) in conjunction with the known effects of depositing at a low pressure[7]. The ZnO:Al was found to have a density of 5g/cm^3, a reduction compared to commonly reported values of 5.45g/cm^3. The density of the deposited SiO_x was found to be 2.15g/cm^3, lower than values expected for crystalline SiO_x confirming the amorphous nature of the films. The a-IGZO/SiO_x superlattices were found to have a low roughness of 0.5 nm at the a-IGZO and SiO_x interfaces. ZnO:Al/SiO_x superlattices were found to have a higher interface roughness of 1 nm. No change in density or surface roughness was observed between reference films and the superlattices. The oscillations arising from the total >150 nm thickness of the deposited superlattice are beyond the threshold resolvable by our system. As a result, the measured pattern consists of only signal from the individual layers allowing for significant reduction in the complexity of the fits. Illustrative examples of the fits obtained are given in Figure S1. The parameters extracted from the fits are given in Supplement S7.

S2. X-Ray Diffraction measurements of the superlattices

Representative X-Ray Diffraction (XRD) patterns of the superlattices are plotted in Figure S2. The reference a-IGZO film and superlattices are found to be fully amorphous regardless of SiO_x thickness. The ZnO:Al reference film and superlattices were all found to be highly textured along the [002] direction. This is consistent with previous results that demonstrated that the inclusion

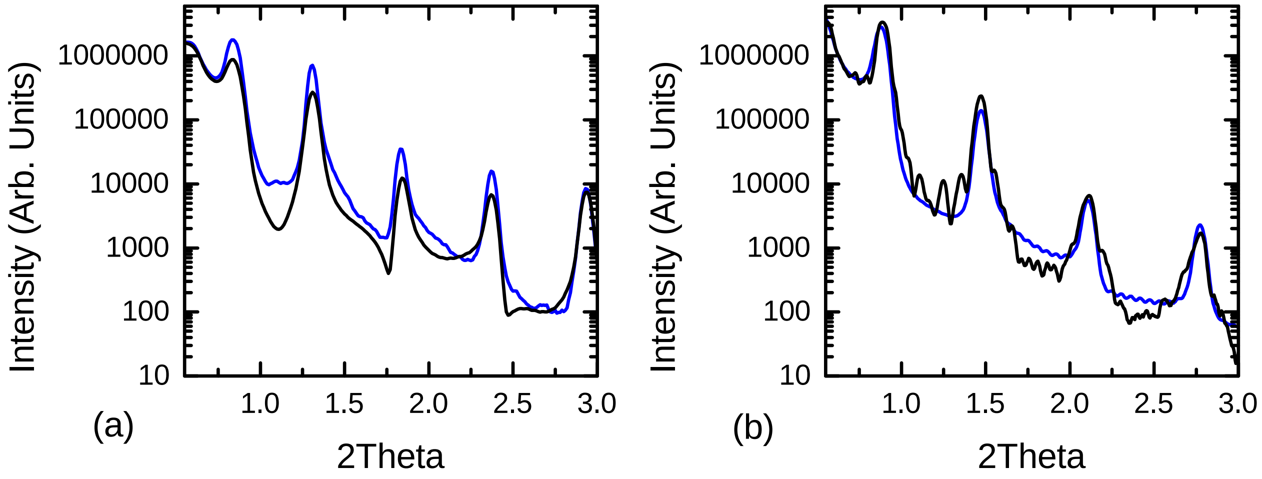

FIG. S1. | XRR fits of the deposited superlattices. XRR fits of the superlattices. **(a)** 10/**5 nm** a-IGZO/SiO_x **(b)** 10/**5 nm** ZnO:Al/SiO_x. (−) Gives the measured data. (−) Gives simulated data.

of a SiO_x buffer layer can serve to reset the crystallographic texture of the growing ZnO:Al film[8]. The dependence of the coherent domain size, as calculated by the Scherrer equation, on ZnO:Al thickness is plotted in the inset of Figure S2. The crystallite size in the 80 nm ZnO:Al film was on the order of 23 nm, while in the superlattices it was found to be on the order of the thickness of the ZnO:Al layer. This reduced crystallite size results from the vertical spacial confinement of the layers in the superlattices. No crystalline peaks from the SiO_x was observed in any superlattice.

S3. a-IGZO Composition

The composition of the a-IGZO target was measured via Energy Dispersive X-ray spectroscopy (EDX) and was found to have a In:Ga:Zn ratio of 1 : 1 : 1. For the thin films, measurements (not shown here) of the In3d, Ga2p and Zn2p peaks were performed in order to obtain the composition of the a-IGZO. Films were found to be highly indium enriched, with the top contact of the deposited superlattices possessing a In:Ga:Zn ratio of 1 : 0.06 : 0.06. The limited penetration depth of XPS measurements ($\approx$ 5 nm) precluded the direct measurement of the In:Ga:Zn ratios of the sub-surface layers. Thus, in order to verify that no change in composition was occurring within the superlattice, the composition of a 10 nm a-IGZO film was compared to the final deposited layer of the superlattices. The variation was found to be negligible, indicating that composition remains consistent throughout the superstructure. This consistency is likely a result of our deposition methodology in which the power applied to the a-IGZO target was ramped down prior to the

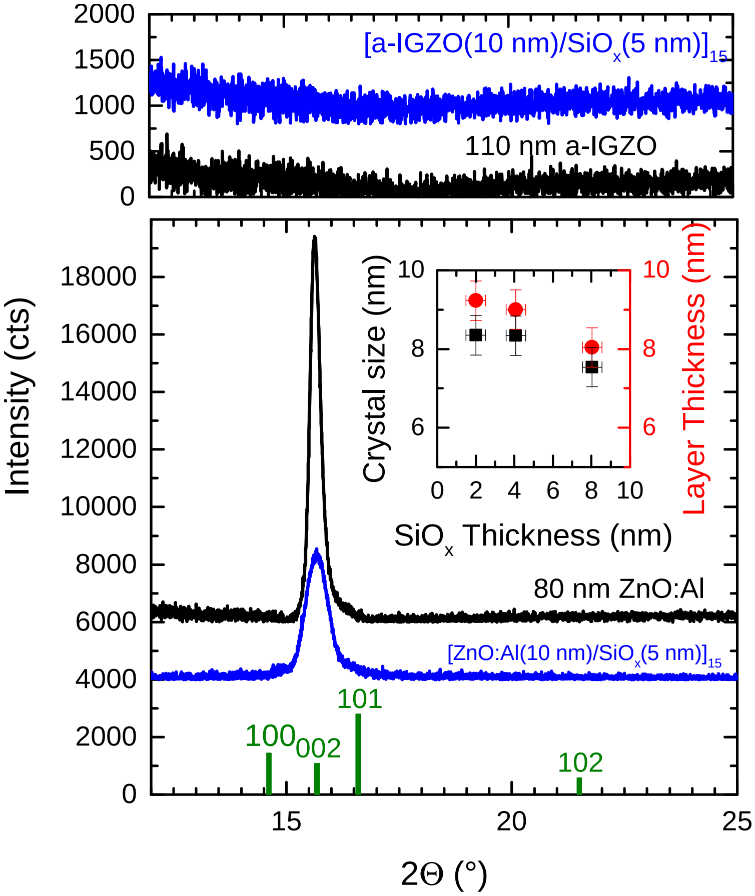

FIG. S2. | XRD of the deposited ZnO:Al and a-IGZO superlattices. XRD patterns of the deposited reference films and superlattices. The upper graph gives the XRD pattern of a (–) 110 nm a-IGZO film and (–) 10/**5 nm** a-IGZO/SiO$_x$ superlattice. The lower graph gives the XRD pattern of the ZnO:Al film and a representative ZnO:Al superlattice. The ZnO:Al is found to be highly textured along the [002] reflex while the a-IGZO is amorphous. (–) 80 nm ZnO:Al(–) 10/**5 nm** ZnO:Al/SiO$_x$ superlattice. Inset gives the ZnO:Al crystallite size as a function of the SiO$_x$ layer thickness (■). Both films possess an amorphous character. The individual ZnO:Al layer thickness obtained from XRR is plotted for comparison (●).

deposition of each layer, reseting the deposition conditions. A reduction in the indium enrichment and increase in the Ga content was detected in the 50 nm a-IGZO film, which possesses a In:Ga:Zn ratio of 1 : 0.18 : 0.06. While the mobility of a-IGZO is known to depend on the composition of the samples[9,10], little variation is observed as a result of this change.

S4. Experimental verification of homogeneous current distribution throughout the superstructure

In order to experimentally confirm that the SiO_x layers were not inhibiting the flow of current to the lower layers of the superlattices, two samples of identical SiO_x layer thicknesses but of different overall thicknesses were deposited for comparison. Two 10/10nm a-IGZO/SiO_xsuperlattices were grown and compared to a 10nm a-IGZO film. The first was 90nm thick overall while the second was 190nm thick. 10nm SiO_x layers were employed as that is the maximum thickness used in this work. Should the current be injected into the full superstructure, it would be expected that the resistivities of the two structures should be the same. Similarly, it would be expected that the sheet resistance of the superlattices would be inversely proportional to film thickness. Results differing from this would indicate that not all of the a-IGZO layers contribute equally to the electron transport and thus that current is not flowing through the full structure. The samples were deposited and annealed in the same manner as the other superlattices. The sheet resistance and resistivity of the deposited superlattices and reference 10nm film are given in Figure S3. It can be observed that the sheet resistance of the 90nm superlattice is half that of the 190nm superlattice leading to similar resistivity. In addition both superlattices display a sheet resistance different than that of a single 10nm a-IGZO film. This verifies that the SiO_x layers are do not appreciably inhibit current flow into the lower layers.

S5. Finite Element Method Simulations

Finite Element Method (FEM) simulations were performed in order to assess the conditions under which the low refractive index layer might inhibit the current distribution throughout the superstructure. The system can be modeled simply in two dimensions, treating the direction along the gold contacts as infinite. A 1 mm wide probe distance was simulated for a 10/10 nm periodicity superlattice film. The resistivity of the a-IGZO layers was selected as $6 \times 10^{-3}\,\Omega$cm and the SiO_x layers were varied in resistivity. An input boundary current was injected at the top surface on one end of the sample and allowed to leave through the top surface at the other end. Figure S4 gives the simulated current density distribution for a 10/10 a-IGZO/SiO_x superlattice for a variety of SiO_x resistivities. Little variation in the current density of the lower layers is observed below $10^7\,\Omega cm$. At SiO_x resistivities above $10^7\,\Omega cm$ the current is preferentially driven through the surface layer with smaller portions traveling through the subsequent a-IGZO layers. Current density is reduced

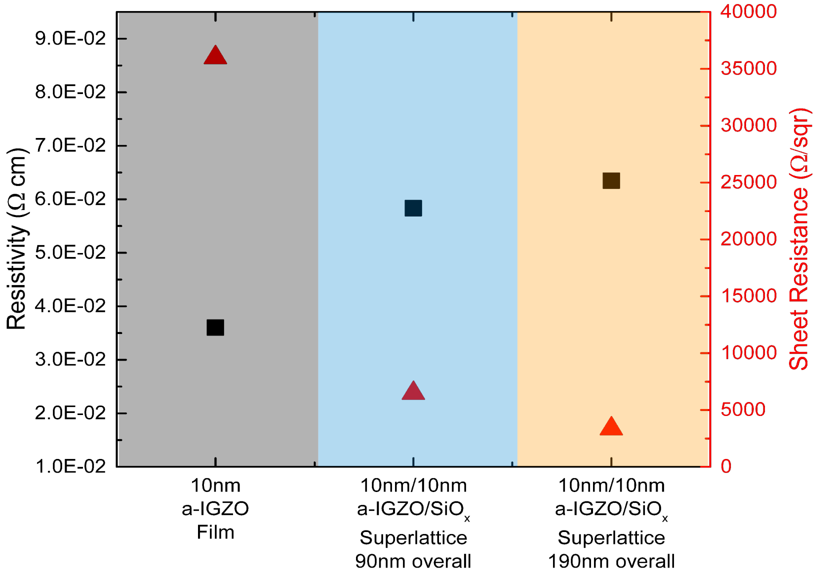

FIG. S3. | Comparison of 10/10 a-IGZO/SiO$_x$ superlattices grown to different overall thicknesses Sheet resistance and resistivity of a 10nm a-IGZO film and two superlattices of identical SiO$_x$ layer thickness grown to different total thicknesses. Shaded Grey area gives the 10nm a-IGZO film. Shaded blue area gives the 90nm total thickness superlattice. Shaded orange gives the 190nm superlattice. (■) Resistivity. (▲) Sheet resistance.

to close to zero within the SiO$_x$ layers. Thus the simulation indicates that provided the SiO$_x$ layer exhibits a resistivity below $10^7\Omega cm$ the current density will be homogeneously distributed. This simulation gives the upper limits for the conductivities of the refractive index layer required to maintain homogeneous current distribution throughout the superstructure.

S6. Discussion of optical bandgap and the possibility of confinement effects

A blueshift in the bulk critical point energy of both superlattices can be observed in Figure 4 (Main article). Unfortunately, analysis of the higher energy range of the refractive index is complicated by the limited dynamic range of our ellipsometer. While a shift can also be observed in Tauc plots of the a-IGZO/SiO$_x$ superlattices grown on sapphire, the analysis is complicated by a shift in the structure of the onset of absorption due to the SiO$_x$ inclusion. Furthermore, as discussed in the main body of this work some of the films experience carrier concentration variation with SiO$_x$ layer thickness resulting in the superposition of the Burnstein-moss induced changes with any confinement resultant expansion. It has been shown that the a-IGZO/SiO$_x$ barrier should lead to

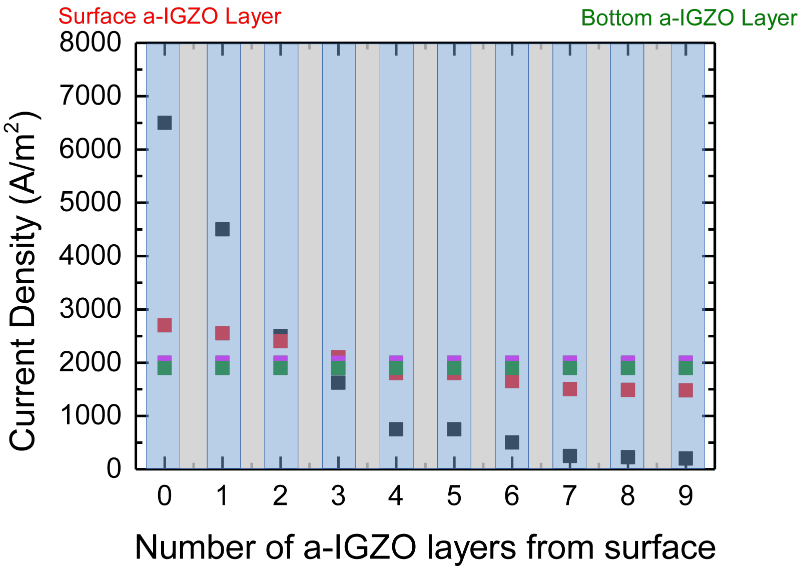

FIG. S4. | Simulation of the current density distribution within a 10/10 a-IGZO/SiO_x superlattice. Plot of the simulated current density in the a-IGZO layers from surface (left) to bottom (right).Graph is shaded blue for the a-IGZO layers and grey for the SiO_x. Current density drops to ≈ 0 for the SiO_x layers. Various resistivities of the SiO_x layers are simulated (■) 10^7 Ωcm, (■) 10^6 Ωcm, (■) 10^5 Ωcm , (■) 10^4 Ωcm

strong confinement[11]. However, experiments performed by Abe et al.[12] indicate that quantisation is unlikely to occur in a-IGZO layers of 10 nm thickness as used in this work. These complications prevent us from drawing a definitive conclusion regarding any possible optical bandgap shifts in the superlattices based on ellipsometry alone.

S7. Structure of the Deposited Superlattices

Tables SI, SII and SIII give the layer thickness, periodicity and electrical properties of the a-IGZO superlattices on glass, a-IGZO superlattices on sapphire and ZnO:Al superlattices on glass respectively. Quoted thicknesses are calculated from the fits of the XRR data.

a-IGZO/SiO_x layer thickness (nm) (nominal)	Number of layers (not including cap)	Total a-IGZO thickness overall (nm)	Total SiO_x thickness overall (nm)	Sheet Resistance (Ω□)	Mobility (cm^2/Vs)
110/0	1	100	0	597	16.5
11/2	15	176	30	9980	10.62
11/3	14	165	42	32000	7.34
11/3.8	13	154	49	25000	6.04
11/4.2	12	143	50	688	14
11/6.8	10	121	68	489	8.57
11/8.3	9	110	74	895	7.877

Table SI. | Composition and electrical properties of the a-IGZO/SiO_x superlattices deposited on glass

a-IGZO/SiO_x layer thickness (nm) (nominal)	Number of layers (not including cap)	Total a-IGZO thickness overall (nm)	Total SiO_xthickness overall (nm)	Sheet Resistance (Ω□)	Mobility (cm^2/Vs)
55/0	1	55	0	1100	16.08
9.8/2.4	15	156	30	4210	10.08
11/4.1	12	143	54	600	9.79
11/8.3	9	110	74	1254	5.96

Table SII. | Composition and electrical properties of the a-IGZO/SiO_x superlattices deposited on Sapphire

$ZnO:Al/SiO_x$ layer thickness (nm) (nominal)	Number of layers (not including cap)	Total ZnO:Al thickness overall (nm)	Total SiO_x thickness overall (nm)	Sheet Resistance ($\Omega\Box$)	Mobility (cm^2/Vs)
81/0	1	81	0	428	7.31
9.2/2	15	148	30	652	5.05
8.9/4	12	116	48	594	4.14
8.2/8	9	82	72	2610	1.01

Table SIII. | Composition and electrical properties of the $ZnO:Al/SiO_x$ superlattices deposited on glass

References

[1] Fullerton, E. E., Schuller, I. K., Vanderstraeten, H. & Bruynseraede, Y. Structural refinement of superlattices from x-ray diffraction. *Phys. Rev. B* **45**, 9292 (1992).

[2] Payne, A. & Clemens, B. Influence of roughness distributions and correlations on x-ray diffraction from superlattices. *Phys. Rev. B* **47**, 2289 (1993).

[3] Barshilia, H. C., Selvakumar, N., Rajam, K., Gopinadhan, K. & Chaudhary, S. Investigation of interface properties of sputter deposited tin/crn superlattices by low angle x-ray reflectivity. *J. Phys. D: Appl. Phys.* **41**, 205409 (2008).

[4] Lamelas, F., He, H. D. & Clarke, R. Numerical modeling of superlattice x-ray-scattering intensities. *Phys. Rev. B* **43**, 12296 (1991).

[5] Huai, Y., Cochrane, R. & Sutton, M. X-ray-diffraction studies of co/re superlattices. *Phys. Rev. B* **48**, 2568–2576 (1993).

[6] Chowdhury, M. D. H., Um, J. G. & Jang, J. Remarkable changes in interface o vacancy and metal-oxide bonds in amorphous indium-gallium-zinc-oxide thin-film transistors by long time annealing at 250 c. *Appl. Phys. Lett.* **105**, 233504 (2014).

[7] Grochowski, J. *et al.* Origin of lower film density and larger defect density in amorphous in–ga–zn–o deposited at high total pressure. *Display Technology, Journal of* **11**, 523–527 (2014).

[8] Owen, J. I., Zhang, W., Köhl, D. & Hüpkes, J. Study on the in-line sputtering growth and structural properties of polycrystalline zno: Al on zno and glass. *J. Cryst. Growth* **344**, 12–18 (2012).

[9] Jeong, J. K. *et al.* High performance thin film transistors with cosputtered amorphous indium gallium zinc oxide channel. *Appl. Phys. Lett.* **91**, 3505 (2007).

[10] Hosono, H. Ionic amorphous oxide semiconductors: Material design, carrier transport, and device application. *J. Non-Cryst. Solids* **352**, 851–858 (2006).

[11] Douglas, E. *et al.* Measurement of $sio_2/inzngao_4$ heterojunction band offsets by x-ray photoelectron spectroscopy. *Appl. Phys. Lett.* **98**, 2110 (2011).

[12] Abe, K., Nomura, K., Kamiya, T. & Hosono, H. Optical evidence for quantization in transparent amorphous oxide semiconductor superlattice. *Phys. Rev. B* **86**, 081202 (2012).
